# Tricyclic antidepressants versus ‘active placebo’, placebo or no intervention for adults with major depressive disorder: a protocol for a systematic review with meta-analysis and Trial Sequential Analysis

**DOI:** 10.1186/s13643-021-01789-0

**Published:** 2021-08-13

**Authors:** Caroline Kamp Jørgensen, Sophie Juul, Faiza Siddiqui, Marija Barbateskovic, Klaus Munkholm, Michael Pascal Hengartner, Irving Kirsch, Christian Gluud, Janus Christian Jakobsen

**Affiliations:** 1 Copenhagen Trial Unit, Centre for Clinical Intervention Research, Copenhagen University Hospital – Rigshospitalet, Blegdamsvej 9, 2100 Copenhagen Ø, Denmark; 2 Stolpegaard Psychotherapy Centre, Mental Health Services in the Capital Region of Denmark, Stolpegaardsvej 28, 2820 Gentofte, Denmark; 3 Department of Psychology, University of Copenhagen, Østre Farimagsgade 2A, 1353 Copenhagen K, Denmark; 4 Cochrane Denmark, Centre for Evidence-Based Medicine Odense, Department of Clinical Research, University of Southern Denmark, JB Winsløwsvej 9b, 5000 Odense, Denmark; 5 Department of Applied Psychology, Zurich University of Applied Sciences, Pfingstweidstrasse 96, 8005 Zurich, Switzerland; 6 Program in Placebo Studies, Harvard Medical School, 330 Brookline Avenue, Boston, MA 02215 USA; 7 Department of Regional Health Research, Faculty of Health Sciences, University of Southern Denmark, J.B. Winsløws Vej 19, 5000 Odense C, Denmark

**Keywords:** Antidepressants, Tricyclic antidepressants, Major depressive disorder, Beneficial effects, Adverse effects

## Abstract

**Background:**

Major depressive disorder is a common psychiatric disorder causing great burden on patients and societies. Tricyclic antidepressants are frequently used worldwide to treat patients with major depressive disorder. It has repeatedly been shown that tricyclic antidepressants reduce depressive symptoms with a statistically significant effect, but the effect is small and of questionable clinical importance. Moreover, the beneficial and harmful effects of all types of tricyclic antidepressants have not previously been systematically assessed. Therefore, we aim to investigate the beneficial and harmful effects of tricyclic antidepressants versus ‘active placebo’, placebo or no intervention for adults with major depressive disorder.

**Methods:**

This is a protocol for a systematic review with meta-analysis that will be reported as recommended by Preferred Reporting Items for Systematic Reviews and Meta-Analysis Protocols, bias will be assessed with the Cochrane Risk of Bias tool—version 2, our eight-step procedure will be used to assess if the thresholds for clinical significance are crossed, Trial Sequential Analysis will be conducted to control random errors and the certainty of the evidence will be assessed with the Grading of Recommendations Assessment, Development and Evaluation approach. To identify relevant trials, we will search both for published and unpublished trials in major medical databases and trial registers, such as CENTRAL, MEDLINE, EMBASE and ClinicalTrials.gov from their inception to 12 May 2021. Clinical study reports will be applied for from regulatory authorities and pharmaceutical companies. Two review authors will independently screen the results from the literature searches, extract data and perform risk of bias assessment. We will include any published or unpublished randomised clinical trial comparing tricyclic antidepressants with ‘active placebo’, placebo or no intervention for adults with major depressive disorder. The following interventions will be assessed: amineptine, amitriptyline, amoxapine, butriptyline, cianopramine, clomipramine, desipramine, demexiptiline, dibenzepin, dosulepin, dothiepin, doxepin, imipramine, iprindole, lofepramine, maprotiline, melitracen, metapramine, nortriptyline, noxiptiline, opipramol, protriptyline, tianeptine, trimipramine and quinupramine. Primary outcomes will be depressive symptoms, serious adverse events and quality of life. Secondary outcomes will be suicide or suicide-attempts and non-serious adverse events. If feasible, we will assess the intervention effects using random-effects and fixed-effect meta-analyses.

**Discussion:**

Tricyclic antidepressants are recommended by clinical guidelines and frequently used worldwide in the treatment of major depressive disorder. There is a need for a thorough systematic review to provide the necessary background for weighing the benefits against the harms. This review will ultimately inform best practice in the treatment of major depressive disorder.

**Systematic review registration:**

PROSPERO CRD42021226161.

**Supplementary Information:**

The online version contains supplementary material available at 10.1186/s13643-021-01789-0.

## Background

### Description of the condition

Major depressive disorder is a psychiatric condition characterised by depressed mood and diminished interest or pleasure [1]. Major depressive disorder is associated with cognitive deficits leading to functional and occupational impairment [2]. The prevalence of major depressive disorder is estimated to be more than 264 million people globally, making it one of the leading contributors to functional disability [3]. Additionally, the high prevalence of major depressive disorder leads to an extensive economic burden estimated at more than 210 billion US dollars annually in the US alone, deriving from direct medical costs as well as costs related to occupational disability and comorbidities [4]. Furthermore, major depressive disorder is associated with an increased risk of suicidal behaviour, with an estimated 15% of patients having attempted suicide at least once in their lifetime [5–7].

### Description of interventions

Tricyclic antidepressants are a group of first-generation antidepressants commonly used for treating major depressive disorder, obsessive–compulsive disorder and chronic pain [8, 9]. The first tricyclic antidepressant, imipramine, was developed in the 1950s by modifying the phenothiazine ring and substituting sulphur with an ethylene bridge [9]. The majority of tricyclic antidepressants function as serotonin and norepinephrine reuptake reuptake inhibitors [10, 11]. By blocking the reuptake of monoamine neurotransmitters in the presynaptic neuron, tricyclic antidepressants theoretically increase the levels of serotonin and norepinephrine in the synaptic cleft [10, 12]. However, the role of monoamines in major depression is unclear and the exact mechanism of action of tricyclic antidepressants is uncertain [10, 13–15].

Whilst selective serotonin reuptake inhibitors are generally recommended as first-line treatment for major depressive disorder, tricyclic antidepressants are amongst recommended treatments for patients whose condition does not improve after treatment with newer medications [16–18]. The World Health Organisation Model List of Essential Medicines includes the tricyclic antidepressant amitriptyline as one of just two essential antidepressants for the treatment of major depressive disorder [19].

### Why is it important to do this review?

Several systematic reviews with meta-analysis have assessed the beneficial effects of tricyclic antidepressants and have concluded that tricyclic antidepressants reduce depressive symptoms with a statistically significant effect for patients with major depressive disorder [20–23]. Some systematic reviews have concluded that tricyclic antidepressants, either as a drug class [20] or as an individual drug [21], are indeed the most effective antidepressants [20, 21]. However, the effect sizes of tricyclic antidepressants were small and may not be important to the average patient [24]. Furthermore, trials comparing antidepressants with ‘active placebo’ (a placebo that mimics the adverse effects of the experimental intervention) indicate that the beneficial effects may in fact be inflated due to the unblinding effects of using an inert placebo [25].

Tricyclic antidepressants are associated with a broad spectrum of adverse effects, but the serious and non-serious adverse events associated with all types of tricyclic antidepressants have not been systematically assessed in adults with major depressive disorder. A recent network meta-analysis published in The Lancet in 2018 included placebo-controlled and head-to-head trials to assess the effects of 21 commonly used antidepressants, including two tricyclic antidepressants, amitriptyline and clomipramine [21]. The results showed that antidepressants compared with placebo seemed to reduce depressive symptoms with a statistically significant effect (standardised mean difference (SMD) 0.30, 95% credibility interval 0.26 to 0.34) [21]. The results also showed that amitriptyline was the most effective antidepressant for reducing depressive symptoms (odds ratio (OR) 2.30, 95% credibility interval 1.89 to 2.41), and that clomipramine was one of the least effective antidepressants for reducing depressive symptoms in the meta-analysis (OR 1.49, 95% credibility interval 1.21 to 1.85) [21]. However, neither serious nor non-serious adverse events were assessed. Instead, the authors assessed lack of ‘acceptability’ (treatment discontinuation measured by the proportion of participants who withdrew for any reason) and the proportion of participants who dropped out early because of adverse effects [21]. Such data on withdrawals as surrogate markers for safety or tolerability should, however, be interpreted with caution due to a number of issues that include difficulty attributing reasons for discontinuation, pressures on patients and investigators to reduce the number of withdrawals, and unblinding that often precedes decisions to withdraw [26].

A Cochrane review published in 2003 investigated effects of low dosage tricyclic antidepressants compared with placebo or standard dosage tricyclic antidepressants in the acute-phase treatment of depressive disorder [23]. Thirty-five trials (2013 participants) compared low dosage tricyclic antidepressants with placebo, and six trials (551 participants) compared low dosage tricyclic antidepressants with standard dosage tricyclic antidepressants [23]. The authors found that low dosage tricyclic antidepressants were more effective in reducing depressive symptoms than placebo, and that standard dosage tricyclic antidepressants were not significantly more effective in reducing depressive symptoms compared with low dosage tricyclic antidepressants [23]. Low dosage tricyclic antidepressants were found to be more likely than placebo to cause at least one adverse effect, and standard dosage was more likely than low dosage tricyclic antidepressants to cause dropouts due to adverse effects [23]. Serious adverse events, suicides and suicide attempts were not assessed. Additionally, this review did not compare standard dosage tricyclic antidepressants with placebo, and not all types of tricyclic antidepressants were included [23].

A meta-analysis of 15 randomised clinical trials published in 2005 assessed the efficacy and tolerability of tricyclic antidepressants and selective serotonin reuptake inhibitors compared with placebo for treatment of depression in primary care [22]. The results showed that tricyclic antidepressants compared with placebo reduced depressive symptoms with a statistically significant effect (SMD − 0.42, 95% confidence interval − 0.55 to − 0.30) [22]. The authors also found that tricyclic antidepressants increased the risk of withdrawal from the trial due to drug-related adverse events [22]. However, the meta-analysis only assessed drug-related adverse events (adverse reactions) and did not assess all adverse effects including serious adverse events. Furthermore, the risk of suicide and suicide attempts were not assessed, and the meta-analysis was limited by only including trials in a primary care setting [22].

Given the limitations of extant systematic reviews, we aim to investigate the beneficial effects and serious and non-serious adverse effects of tricyclic antidepressants for major depressive disorder in adults including both published and unpublished data. Our systematic review will take bias risk (systematic errors), play of chance (random errors) and certainty of the findings into consideration. This systematic review will be conducted as part of a larger project investigating the beneficial and harmful effects of all antidepressants for major depressive disorder [27]. In addition to this systematic review, we will also publish separate systematic reviews for selective serotonin reuptake inhibitors, duloxetine [28], venlafaxine and mirtazapine [27]. These systematic reviews will ultimately provide data for a systematic review investigating the effects of all antidepressants for major depressive disorder [27]. We chose to publish the present protocol and systematic review separately to investigate the effects of tricyclic antidepressants in more detail (i.e. more outcomes) [27].

## Methods

The present protocol has been registered in the PROSPERO database (CRD42021226161) and is reported in accordance with the reporting guidance provided in the Preferred Reporting Items for Systematic Reviews and Meta-Analysis Protocols (PRISMA-P) statement [29, 30] (see checklist in Additional file 1).

### Criteria for considering trials for this review

#### Types of trials

We will include randomised clinical trials irrespective of trial design, setting, publication status, publication year and language. We will not include quasi-randomised trials, cluster-randomised trials or non-randomised studies, as they are at greater risk of bias. By excluding such studies and trials we are, however, aware that we may miss some data on adverse effects, especially rare and late occurring adverse events.

#### Types of participants

Adults (as defined by trialists) with a primary diagnosis of major depressive disorder as defined by standardised diagnostic criteria such as the Diagnostic and Statistical Manual of Mental Disorders, Fifth Edition [1], International Classification of Diseases, 10th Revision [31] or earlier versions of these diagnostic manuals. Major depressive disorder must be the primary diagnosis, and we will therefore not include trials randomising participants with a primary somatic diagnosis and comorbid major depressive disorder. Participants will be included irrespective of sex and comorbidities. If a trial reports data where only a subset of participants is eligible (e.g. a combination of adolescents and adults), we will only include those that fulfil the inclusion criteria, and it therefore requires that data can be obtained for that specific group.

#### Types of interventions

As experimental intervention, we will include the following tricyclic antidepressants: amineptine, amitriptyline, amoxapine, butriptyline, cianopramine, clomipramine, desipramine, demexiptiline, dibenzepin, dosulepin, dothiepin, doxepin, imipramine, iprindole, lofepramine, maprotiline, melitracen, metapramine, nortriptyline, noxiptiline, opipramol, protriptyline, tianeptine, trimipramine and quinupramine [32] irrespective of dose and duration of administration. We will only include treatment arms that use doses within the licenced dose range.

As control intervention, we will include: ‘active placebo’ (a matching placebo that produces noticeable and comparable adverse effects to tricyclic antidepressants that may convince the participant and blinded outcome assessors that the participants are receiving an ‘active’ intervention), placebo or no intervention, e.g. ‘waiting-list’.

#### Cointerventions

We will accept any co-intervention (e.g. other drug treatment or psychotherapy), if the co-intervention is planned to be delivered similarly in the intervention and control groups.

### Outcome measures

#### Primary outcomes

1. Depressive symptoms measured on the 17-item or 21-item Hamilton Depression Rating Scale (HDRS) [33]. Where the 21-item scale is used, we will only include the data if the total score is only based on the first 17 items.
2. The proportion of participants with one or more serious adverse events. We will use the International Conference on Harmonization of technical requirements for registration of pharmaceuticals for human use—Good Clinical Practice (ICH-GCP) definition of a serious adverse event, which is any untoward medical occurrence that resulted in death, was life-threatening, required hospitalisation or prolonging of existing hospitalisation and resulted in persistent or significant disability or jeopardised the participant [34]. If the trialists do not use the ICH-GCP definition, we will include the data if the trialists use the term ‘serious adverse event’. If the trialists do not use the ICH-GCP definition nor use the term serious adverse event, then we will also include the data provided the event clearly fulfils the ICH-GCP definition for a serious adverse event. We will secondly assess each serious adverse event separately (see below).
3. Quality of life (any valid continuous scale, e.g. the EQ-5D [35])

#### Secondary outcomes

1. The proportion of participants with either a suicide or a suicide-attempt (as defined by the trialists).
2. The proportion of participants with one or more non-serious adverse events (any adverse event not classified as serious). We will secondly assess each non-serious adverse event separately (see below).

#### Exploratory outcomes

1. Depressive symptoms measured on the Montgomery-Asberg Depression Rating Scale (MADRS) [36], the Beck’s Depression Inventory (BDI) [37], or the 6-item HDRS [38].
2. Individual serious adverse events.
3. Individual non-serious adverse events.
4. Suicidal ideation (any valid continuous scale).
5. The proportion of participants achieving response. We have defined response as a 50% reduction (from baseline) on either HDRS, MADRS or any other scale as used by trialists.
6. The proportion of participants achieving remission as defined by trialists.

#### Assessment time points

We will assess all our outcomes at end of treatment primarily and at maximum follow-up secondarily.

### Search methods for identification of trials

#### Electronic searches

We will search the Cochrane Central Register of Controlled Trials (CENTRAL), Medical Literature Analysis and Retrieval System Online (MEDLINE), Excerpta Medica Database (EMBASE), Latin American and Caribbean Health Sciences Literature (LILACS), PsycINFO, Science Citation Index Expanded (SCI-EXPANDED), Social Sciences Citation Index (SSCI), Chinese Biomedical Literature Database (CBM), China Network Knowledge Information (CNKI), Chinese Science Journal Database (VIP), Wafang Database, Conference Proceedings Citation Index—Science (CPCI-S) and Conference Proceedings Citation Index—Social Science & Humanities (CPCI-SSH) to identify relevant trials. We will search all databases from their inception to 12 May 2021. For a detailed search strategy for all electronic databases, see Additional file 2. The search strategies for the Chinese databases will be given at review stage. Trials will be included irrespective of language, publication status, publication year and publication type.

#### Searching other resources

The reference lists of relevant publications will be checked for any unidentified randomised trials. We will contact the authors of included trials by email asking for unpublished randomised trials. To identify unpublished trials, we will also search clinical trial registers (ClinicalTrials.gov and the ICTRP Search Portal [39]), websites of pharmaceutical companies, websites of U.S. Food and Drug Administration (FDA), and European Medicines Agency (EMA). We will request FDA, EMA and national medicines agencies to provide all publicly releasable information about relevant randomised clinical trials of antidepressants that were submitted for marketing approval, including clinical study reports [26]. Additionally, we will hand search conference abstracts from psychiatry conferences for relevant trials. We will also include unpublished and grey literature trials if we identify these and assess relevant retraction statements and errata for included trials.

### Data collection and analysis

We will perform and report the review following the recommendations stated in the Cochrane Handbook for Systematic Reviews of Interventions [26]. Analyses will be performed using Stata version 16.1 (StataCorp LLC, College Station, TX, USA) [40] and Trial Sequential Analysis [41, 42].

#### Selection of trials

Two review authors will independently screen titles and abstracts. We will retrieve all relevant full-text study reports/publications, and two review authors will independently screen the full text to identify and record reasons for exclusion of the ineligible trials. The two review authors will resolve any disagreement through discussion, or, if required, they will consult with a third author.

#### Data extraction and management

Two authors will independently extract data from included trials. Disagreements will be resolved by discussion with a third author. The two review authors will assess duplicate publications and companion papers of a trial together to evaluate all available data simultaneously (maximise data extraction, correct bias assessment). We will contact the trial authors by email to obtain any additional data, which may not have been reported sufficiently or at all in the publication.

#### Trial characteristics

We will extract the following data: bias risk components (as defined below); trial design (parallel, factorial, or crossover); number of intervention groups; length of follow-up; estimation of sample size; inclusion and exclusion criteria; for-profit funding of trial and NCT/EudraCT number.

#### Participant characteristics

We will extract the following data: number of randomised participants; number of analysed participants; number of participants lost to follow-up/withdrawals/crossover; age range (mean and standard deviation) and sex ratio.

#### Intervention characteristics

We will extract the following data: type of tricyclic antidepressant; dose of intervention; duration of intervention.

#### Control characteristics

We will extract the following data: type of control intervention; dose of intervention; duration of intervention.

#### Outcomes

All outcomes listed above will be extracted from each randomised clinical trial, and we will identify if outcomes are incomplete or selectively reported according to the criteria described later in “incomplete outcome data” bias domain and “selective outcome reporting” bias domain.

#### Notes

We will search for information regarding industry funding of either personal or academic activities for each trial author. We will judge a publication at high risk of for-profit bias if a trial is sponsored by the industry (including trials partly sponsored by the industry, e.g. if the trial drug was sponsored by a medical company), or if just one author has any affiliation to the industry. We will note in the ‘characteristics of included studies’ table if outcome data were not reported in a usable way. Two review authors will independently transfer data into the Stata file [40]. Disagreements will be resolved through discussion, or if required, we will consult with a third author.

#### Assessment of risk of bias in the included trials

Our bias risk assessment will be based on the Cochrane Risk of Bias tool—version 2 (RoB 2) as recommended in the Cochrane Handbook of Systematic Reviews of Interventions [26]. We will evaluate the methodology in respect of the following bias domains:

##### Bias arising from the randomisation process

- Low risk of bias: Allocation was adequately concealed, AND baseline imbalances across intervention groups appear to be compatible with chance, AND an adequate (random or otherwise unpredictable) method was used to generate allocation sequence, OR there is no information about the method used to generate the allocation sequence.
- Some concerns: Allocation was adequately concealed, AND there is a problem with the method of sequence generation, OR baseline imbalances suggest a problem with the randomisation process, OR no information is provided about concealment of allocation, AND baseline imbalances across intervention groups appear to be compatible with chance, OR no information to answer any of the signalling questions.
- High risk of bias: Allocation sequence was not concealed, OR no information is provided about concealment of allocation sequence, AND baseline imbalances suggest a problem with the randomisation process.

##### Bias due to deviation from intended interventions

- Low risk of bias: Participants, carers and personnel were unaware of intervention groups during the trial, OR participants, carers or personnel were aware of intervention groups during the trial but any deviations from intended intervention reflected usual practice, OR participants, carers or personnel were aware of intervention groups during the trial but any deviations from intended intervention were unlikely to impact on the outcome, AND no participants were analysed in the wrong intervention groups (that is, on the basis of intervention actually received rather than of randomised allocation).
- Some concerns: Participants, carers or personnel were aware of intervention groups and there is no information on whether there were deviations from usual practice that were likely to impact on the outcome and were imbalanced between intervention groups, OR some participants were analysed in the wrong intervention groups (on the basis of intervention actually received rather than of randomised allocation) but there was little potential for a substantial impact on the estimated effect of intervention.
- High risk of bias: Participants, carers or personnel were aware of intervention groups, and there were deviations from intended interventions that were unbalanced between the intervention groups and likely to have affected the outcome, OR some participants were analysed in the wrong intervention groups (on the basis of intervention actually received rather than of randomised allocation), and there was potential for a substantial impact on the estimated effect of intervention.

##### Bias due to missing outcome data

- Low risk of bias: No missing data OR non-differential missing data (similar proportion of and similar reasons for missing data in compared groups) OR evidence of robustness of effect estimate to missing data (based on adequate statistical methods for handling missing data and sensitivity analysis).
- Some concerns: An unclear degree of missing data or unclear information on proportion and reasons for missingness in compared groups AND there is no evidence that the effect estimate is robust to missing data.
- High risk of bias: A high degree of missing data AND differential missing data (different proportion of or different reasons for missing data in compared groups) AND there is no evidence that the effect estimate is robust to missing data.

##### Bias in measurement of outcomes

- Low risk of bias: The outcome assessors were unaware of the intervention received by study participants, OR the outcome assessors were aware of the intervention received by study participants, but the assessment of the outcome was unlikely to be influenced by knowledge of the intervention received.
- Some concerns: There is no information available to determine whether the assessment of the outcome is likely to be influenced by knowledge of the intervention received.
- High risk of bias: The assessment of the outcome was likely to be influenced by knowledge of the intervention received by study participants.

##### Bias arising from selective reporting of results

- Low risk of bias: Reported outcome data are unlikely to have been selected, on the basis of the results, from multiple outcome measurements (e.g. scales, definitions, time points) within the outcome domain, and reported outcome data are unlikely to have been selected, on the basis of the results, from multiple analyses of the data.
- Some concerns: There is insufficient information available to exclude the possibility that reported outcome data were selected, on the basis of the results, from multiple outcome measurements (e.g. scales, definitions, time points) within the outcome domain, or from multiple analyses of the data. Given that analysis intentions are often unavailable or not reported with sufficient detail, we anticipate that this will be the default judgement for most trials.
- High risk of bias: Reported outcome data are likely to have been selected, on the basis of the results, from multiple outcome measurements (e.g. scales, definitions, time points) within the outcome domain, or from multiple analyses of the data (or both).

##### Overall assessment of risk of bias

- Low risk of bias: The trial is judged to be at low risk of bias for all domains.
- High risk of bias: The trial is judged to be at high risk of bias or to be at some concerns in at least one domain. Our subgroup analysis will compare the intervention effect of trials at low risk of bias with trials at high risk of bias, that is one or more domains at some concern or high risk of bias.

We will assess the domains ‘missing outcome data’, ‘risk of bias in measurement of the outcome’ and ‘risk of bias in selection of the reported result’ for each outcome result. Thus, we can assess the bias risk for each outcome assessed in addition to each trial. Our primary conclusions will be based on the results of our primary outcome results with overall low risk of bias. Both our primary and secondary results will be presented in the ‘Summary of Findings’ tables.

#### Differences between the protocol and the review

We will conduct the review according to this published protocol and report any deviations from it in the ‘Differences between the protocol and the review’ section of the systematic review.

#### Measurement of treatment effect

##### Dichotomous outcomes

We will calculate risk ratios (RRs) with 95% confidence interval (CI) for dichotomous outcomes, as well as the Trial Sequential Analysis-adjusted CIs (see below).

##### Continuous outcomes

We will calculate the mean differences (MDs) and consider calculating the SMD with 95% CI for continuous outcomes. We will also calculate Trial Sequential Analysis-adjusted CIs (see below).

#### Dealing with missing data

We will use intention-to-treat data if provided by the trialists [43]. We will, as the first option, contact all trial authors to obtain any relevant missing data (i.e. for data extraction and for assessment of risk of bias, as specified above).

##### Dichotomous outcomes

We will not impute missing values for any outcomes in our primary analysis. In our sensitivity analyses (see paragraph below), we will impute data.

##### Continuous outcomes

We will primarily analyse scores assessed at single time points. If only changes from baseline scores are reported, we will analyse the results together with follow-up scores [26]. If standard deviations (SDs) are not reported, we will calculate the SDs using trial data, if possible. We will not use intention-to-treat data if the original report did not contain such data. We will not impute missing values for any outcomes in our primary analysis. In our sensitivity analysis (see paragraph below) for continuous outcomes, we will impute data.

#### Assessment of heterogeneity

We will primarily investigate forest plots to visually assess any sign of heterogeneity. We will secondly assess the presence of statistical heterogeneity by chi^2^ test (threshold *P* < 0.10) and measure the quantities of heterogeneity by the *I*^2^ statistic [44, 45]. We will investigate possible heterogeneity through subgroup analyses. We may ultimately decide that a meta-analysis should be avoided [26].

#### Assessment of reporting biases

We will use a funnel plot to assess reporting bias if ten or more trials are included. We will visually inspect funnel plots to assess the risk of small trial effects that could potentially reflect publication bias. We are aware of the limitations of a funnel plot (i.e. a funnel plot assesses bias due to small sample size). From this information, we will assess possible risk of publication bias. For dichotomous outcomes, we will test asymmetry with the Harbord test [46] if *τ*^2^ is less than 0.1 and with the Rücker test if *τ*^2^ is more than 0.1. For continuous outcomes, we will use the regression asymmetry test [47] and the adjusted rank correlation [48].

#### Unit of analysis issues

We will only include randomised clinical trials. For trials using crossover design, only data from the first period will be included [26, 49]. We will not include cluster randomised trials. Where multiple trial arms are reported in a single trial, we will include only the relevant arms. For trials with multiple relevant experimental groups, we will either combine the groups (when considered subtypes of the same intervention) or divide the number of events and sample size of the control group (e.g. for two different types of tricyclic antidepressants). For continuous data, we will keep the main score [26]. In case of, for example, a 2 × 2 factorial design trial, the two groups receiving antidepressants will be considered experimental groups, whilst the two groups receiving ‘active placebo’, placebo or no intervention will be considered control groups.

### Data synthesis

#### Meta-analysis

We will undertake the meta-analysis according to the Cochrane Handbook for Systematic Reviews of Interventions [26], Keus et al. [50] and our eight-step procedure suggested by Jakobsen et al. [51]. We will use the statistical software Stata version 16 to analyse data [40]. We will assess the intervention effects with both random-effects model meta-analyses (Hartung-Knapp-Sidik-Jonkman) [52] and fixed-effect model meta-analyses (Mantel–Haenszel for dichotomous outcomes and inverse variance for continuous outcomes) [26, 53]. We will use the more conservative point estimate of the two [51]. The more conservative point estimate is the estimate with the highest *P* value. We assess a total of five primary and secondary outcomes, and we will therefore consider a *P* value of 0.016 or less as the threshold for statistical significance [51]. We will investigate possible heterogeneity through subgroup analyses. We will use our eight-step procedure to assess if the thresholds for significance are crossed [51]. This eight-step procedure is comprised of the following steps: (1) obtain the 95% confidence intervals and the *P* values from both fixed-effect and random-effects meta-analyses and report the most conservative results as the main results; (2) explore the reasons behind substantial statistical heterogeneity using subgroup and sensitivity analyses (see step 6); (3) to take account of problems with multiplicity adjust the thresholds for significance according to the number of primary outcomes (we will also adjust for secondary outcomes); (4) calculate required information sizes (≈ the a priori required number of participants for a meta-analysis to be conclusive) for all outcomes and analyse each outcome with Trial Sequential Analysis. Report whether the trial sequential monitoring boundaries for benefit, harm or futility are crossed; (5) calculate Bayes factors for all primary outcomes; (6) use subgroup analyses and sensitivity analyses to assess the potential impact of bias on the review results; (7) assess the risk of publication bias; (8) assess the clinical significance of the statistically significant review results [51].

#### Trial sequential analysis

Traditional meta-analysis runs the risk of random errors due to sparse data and repetitive testing of accumulating data when updating reviews. We wish to control the risks of type I and type II errors. We will therefore perform Trial Sequential Analysis on all outcomes, in order to calculate the required information size (that is, the number of participants needed in a meta-analysis to detect or reject a certain intervention effect) and the cumulative Z-curve’s breach of relevant trial sequential monitoring boundaries [41, 42, 54–60]. A more detailed description of Trial Sequential Analysis can be found in the manual [42] and at http://www.ctu.dk/tsa/. For dichotomous outcomes, we will estimate the required information size based on the observed proportion of patients with an outcome in the control group (the cumulative proportion of patients with an event in the control groups relative to all patients in the control groups), a relative risk reduction or a relative risk increase of 20%, an alpha of 1.6% for all our outcomes, a beta of 10% and the observed diversity as suggested by the trials in the meta-analysis. For continuous outcomes, we will in the Trial Sequential Analysis use the observed standard deviation (SD) in the control group, a mean difference of three HDRS points when assessing depressive symptoms; otherwise, the observed SD/2, an alpha of 1.6% for all outcomes, a beta of 10%, and the observed diversity as suggested by the trials in the meta-analysis.

#### Subgroup analysis and integration of heterogeneity

##### Subgroup analysis

We will perform the following subgroup analyses when analysing the primary outcomes (depressive symptoms, serious adverse events, quality of life).

- Trials at high risk of bias compared to trials at low risk of bias
- Trials without for profit bias compared to trials at unknown or known risk of for profit bias [61]
- Types of tricyclic antidepressant agents (amineptine, amitriptyline, amoxapine, butriptyline, cianopramine, clomipramine, desipramine, demexiptiline, dibenzepin, dosulepin, dothiepin, doxepin, imipramine, iprindole, lofepramine, maprotiline, melitracen, metapramine, nortriptyline, noxiptiline, opipramol, protriptyline, tianeptine, trimipramine and quinupramine)
- Types of comparator (‘active placebo’, placebo no intervention)
- Age groups (18 to 24 years, 25 to 64 years, ≥ 65 years)
- Type of definition used for serious adverse events. This may be the ICH-GCP definition, the term ‘serious adverse events’, or data that clearly fulfils the ICH-GCP definition but is not referred to by the abovementioned definitions.
- Type of diagnostic criteria (operationalised criteria versus non-operationalised criteria).

We will use the formal test for subgroup interactions in Stata [40].

##### Sensitivity analysis

To assess the potential impact of the missing data for dichotomous outcomes, we will perform the two following sensitivity analyses on both the primary and secondary dichotomous outcomes.

‘Best–worst-case’ scenario: We will assume that all participants lost to follow-up in the antidepressant group survived, had no serious adverse events, had no suicides or suicide attempts and had no non-serious adverse events, and that all those participants lost to follow-up in the control group did not survive, had a serious adverse event, died by suicide or had a suicide attempt and had a non-serious adverse event.

‘Worst-best-case’ scenario: We will assume that all participants lost to follow-up in the antidepressant group did not survive, had a serious adverse event, died by suicide or had a suicide attempt and had a non-serious adverse event, and that all those participants lost to follow-up in the control group survived, had no serious adverse events, had no suicides or suicide attempts and had no non-serious adverse events.

We will present results of both scenarios in our review. When analysing depressive symptoms and quality of life, a ‘beneficial outcome’ will be the group mean plus two SDs (we will secondly use one SD in another sensitivity analysis) of the group mean and a ‘harmful outcome’ will be the group mean minus two SDs (we will secondly use one SD in another sensitivity analysis) of the group mean [51]. To assess the potential impact of missing SDs for continuous outcomes, we will perform the following sensitivity analysis:

Where SDs are missing and it is not possible to calculate them, we will impute SDs from trials with similar populations and low risk of bias. If we find no such trials, we will impute SDs from trials with a similar population. As the final option, we will impute the mean SD from all included trials.

We will present results of this scenario in our review. Other post hoc sensitivity analyses might be warranted if unexpected clinical or statistical heterogeneity is identified during the analysis of the review results [51].

##### Summary of findings table

We will create a summary of findings table for each comparison (tricyclic antidepressants vs. ‘active placebo’, placebo and no intervention) including each of the prespecified primary and secondary outcomes (depressive symptoms, serious adverse events, quality of life, suicides or suicide attempts, non-serious adverse events). We will use the Grading of Recommendations, Assessment, Development and Evaluations (GRADE) considerations (bias risk, heterogeneity, imprecision, indirectness and publication bias) to assess the quality of a body of evidence [51, 62–64]. We will assess imprecision using Trial Sequential Analysis. We will justify all decisions to downgrade the quality of evidence using footnotes, and we will make comments to aid the reader’s understanding of the review where necessary. Firstly, we will present our results in the summary of findings table based on the results from the trials with overall low risk of bias, and secondly, we will present the results based on all trials.

## Discussion

This protocol aims to assess the beneficial and harmful effects of tricyclic antidepressants versus ‘active placebo’, placebo or no intervention in adults with major depressive disorder. Primary outcomes will be depressive symptoms, serious adverse events and quality of life. Secondary outcomes will be suicide or suicide attempts, and non-serious adverse events.

Our protocol has several strengths. The predefined methodology is based on Cochrane methodology [26], Keus et al. [50], our eight-step assessment suggested by Jakobsen et al. [51], Trial Sequential Analysis [41] and GRADE assessment [62–64]. Hence, this protocol considers both risks of random errors and risks of systematic errors as well as risks of external validity, heterogeneity and risks of publication bias [51]. Furthermore, we increase the statistical power by pooling all tricyclic antidepressants as the experimental intervention. This inclusiveness also allows us to assess the different tricyclic antidepressants relative effects to the comparators. Moreover, we will include data from both unpublished and published trials as well as clinical study reports [26]. The latter should secure a fairer comparison of benefits and harms [26].

Our protocol also has limitations. The primary limitation is the potential for high statistical heterogeneity due to the inclusion of various tricyclic antidepressants as the experimental intervention. To minimise this limitation, we will carefully look for signs of heterogeneity and ultimately decide if data ought to be meta-analysed, and we have planned several sensitivity analyses and subgroup analyses. Another limitation is the large number of comparisons which increases the risks of type 1 errors. We have adjusted our thresholds for significance according to the number of primary and secondary outcomes, but we have not adjusted our thresholds for significance according to the total number of comparisons (e.g. subgroup analyses and sensitivity analyses). Moreover, we expect inadequate reporting of harmful effects in the included trials, which increases the risk of underestimation of harmful effects [26]. Although we will request unpublished randomised trials, we expect challenges with obtaining the unpublished data. Finally, we expect short treatment and follow-up periods which may not accurately mimic how antidepressants are used in clinical practice [65, 66].

Although tricyclic antidepressants have previously been investigated in systematic reviews, no former review has systematically assessed the beneficial and harmful effects of all types of tricyclic antidepressants compared with ‘active placebo’, placebo or no intervention. Since tricyclic antidepressants are recommended by clinical guidelines and frequently used worldwide [17, 19, 67], there is a need for a systematic review assessing the benefits and the harms in treatment of adults with major depressive disorder. The review will ultimately inform best practice in the treatment of major depressive disorder.

## Supplementary Information

**Additional file 1.** PRISMA-P (Preferred Reporting Items for Systematic review and Meta-Analysis Protocols) 2015 checklist: recommended items to address in a systematic review protocol*.

**Additional file 2.** Search strategies for ‘Tricyclic antidepressants for major depressive disorder’.

## Data Availability

Not applicable.

## Abbreviations

BDI: Beck’s Depression Inventory
CBM: Chinese Biomedical Literature Database
CENTRAL: Cochrane Central Register of Controlled Trials
CI: Confidence interval
CNKI: China Network Knowledge Information
CPCI-S: Conference Proceedings Citation Index—Science
CPCI-SSH: Conference Proceedings Citation Index—Social Science & Humanities
EMA: European Medicines Agency
EMBASE: Excerpta Medica Database
FDA: US Food and Drug Administration
GRADE: Grading of Recommendations, Assessment, Development and Evaluation
HDRS: Hamilton Depression Rating Scale
ICH-GCP: Good Clinical Practice
LILACS: Latin American and Caribbean Health Sciences Literature
MADRS: Montgomery-Asberg Depression Rating Scale
MDs: Mean differences
MEDLINE: Medical Literature Analysis and Retrieval System Online
OR: Odds ratio
PRISMA-P: Preferred Reporting Items for Systematic Reviews and Meta-Analysis Protocols
RoB 2: Cochrane Risk of Bias tool—version 2
RRs: Risk ratios
SCI-EXPANDED: Science Citation Index Expanded
SDs: Standard deviations
SMD: Standardised mean difference
SSCI: Social Sciences Citation Index
VIP: Chinese Science Journal Database
WHO: World Health Organisation
